# Plant diversity in sedimentary DNA obtained from high-latitude (Siberia) and high-elevation lakes (China)

**DOI:** 10.3897/BDJ.8.e57089

**Published:** 2020-12-14

**Authors:** Kathleen Rosmarie Stoof-Leichsenring, Sisi Liu, Weihan Jia, Kai Li, Luidmila A. Pestryakova, Steffen Mischke, Xianyong Cao, Xingqi Liu, Jian Ni, Stefan Neuhaus, Ulrike Herzschuh

**Affiliations:** 1 Polar Terrestrial Environmental Systems, Alfred Wegener Institute Helmholtz Centre for Polar and Marine Research, Potsdam, Germany Polar Terrestrial Environmental Systems, Alfred Wegener Institute Helmholtz Centre for Polar and Marine Research Potsdam Germany; 2 Institute of Environmental Science and Geography, University of Potsdam, Potsdam, Germany Institute of Environmental Science and Geography, University of Potsdam Potsdam Germany; 3 College of Resource Environment and Tourism, Capital Normal University, Beijing, China College of Resource Environment and Tourism, Capital Normal University Beijing China; 4 College of Chemistry and Life Sciences, Zhejiang Normal University, Jinhua, China College of Chemistry and Life Sciences, Zhejiang Normal University Jinhua China; 5 Department for Geography and Biology, North-Eastern Federal University of Yakutsk, Yakutsk, Russia Department for Geography and Biology, North-Eastern Federal University of Yakutsk Yakutsk Russia; 6 Institute of Earth Sciences, University of Iceland, Reykjavík, Iceland Institute of Earth Sciences, University of Iceland Reykjavík Iceland; 7 Alpine Paleoecology and Human Adaptation Group (ALPHA), Key Laboratory of Alpine Ecology, Institute of Tibetan Plateau Research, Beijing, China Alpine Paleoecology and Human Adaptation Group (ALPHA), Key Laboratory of Alpine Ecology, Institute of Tibetan Plateau Research Beijing China; 8 Computing and Data Centre, Alfred Wegener Institute Helmholtz Centre for Polar and Marine Research, Bremerhaven, Germany Computing and Data Centre, Alfred Wegener Institute Helmholtz Centre for Polar and Marine Research Bremerhaven Germany; 9 Institute of Biochemistry and Biology, University of Potsdam, Potsdam, Germany Institute of Biochemistry and Biology, University of Potsdam Potsdam Germany

**Keywords:** Arctic, chloroplast DNA, lakes, metabarcoding, plant diversity, sedimentary DNA, Tibet Plateau, trnL P6 loop, vegetation

## Abstract

**Background:**

Plant diversity in the Arctic and at high altitudes strongly depends on and rebounds to climatic and environmental variability and is nowadays tremendously impacted by recent climate warming. Therefore, past changes in plant diversity in the high Arctic and high-altitude regions are used to infer climatic and environmental changes through time and allow future predictions. Sedimentary DNA (sedDNA) is an established proxy for the detection of local plant diversity in lake sediments, but still relationships between environmental conditions and preservation of the plant sedDNA proxy are far from being fully understood. Studying modern relationships between environmental conditions and plant sedDNA will improve our understanding under which conditions sedDNA is well-preserved helping to a.) evaluate suitable localities for sedDNA approaches, b.) provide analogues for preservation conditions and c.) conduct reconstruction of plant diversity and climate change. This study investigates modern plant diversity applying a plant-specific metabarcoding approach on sedimentary DNA of surface sediment samples from 262 lake localities covering a large geographical, climatic and ecological gradient. Latitude ranges between 25°N and 73°N and longitude between 81°E and 161°E, including lowland lakes and elevated lakes up to 5168 m a.s.l. Further, our sampling localities cover a climatic gradient ranging in mean annual temperature between -15°C and +18°C and in mean annual precipitation between 36­ and 935 mm. The localities in Siberia span over a large vegetational gradient including tundra, open woodland and boreal forest. Lake localities in China include alpine meadow, shrub, forest and steppe and also cultivated areas. The assessment of plant diversity in the underlying dataset was conducted by a specific plant metabarcoding approach.

**New information:**

We provide a large dataset of genetic plant diversity retrieved from surface sedimentary DNA from lakes in Siberia and China spanning over a large environmental gradient. Our dataset encompasses sedDNA sequence data of 259 surface lake sediments and three soil samples originating from Siberian and Chinese lakes. We used the established chloroplastidal P6 loop trnL marker for plant diversity assessment. The merged, filtered and assigned dataset includes 15,692,944 read counts resulting in 623 unique plant DNA sequence types which have a 100% match to either the EMBL or to the specific Arctic plant reference database. The underlying dataset includes a taxonomic list of identified plants and results from PCR replicates, as well as extraction blanks (BLANKs) and PCR negative controls (NTCs), which were run along with the investigated lake samples. This collection of plant metabarcoding data from modern lake sediments is still ongoing and additional data will be released in the future.

## Introduction

Arctic and high-elevation ecosystems are very sensitive to natural and anthropogenically-induced climate variability. Anthropogenic warming and changes in land-use have been considered to shift vegetation composition and plant richness in these areas during the last centuries and decades (Cui and Graf 2009, Xu and Liu 2007, Pearson et al. 2013). Still, there is a lack of understanding how environmental variability shaped local plant diversity in these remote areas. Modern plant sedDNA data, as a modern training-set, can be applied to reconstruct past vegetation types in alpine and arctic ecosystems, which in pollen-based reconstruction, can be largely biased due to the presence of poor modern pollen analogues (Ortu 2010). Since the last decade, plant sedDNA have been applied to lake surface-sediment samples to identify modern plant communities (Alsos et al. 2018, Niemeyer et al. 2017) and to lake sediment cores to reconstruct local plant diversity changes in the past (Clarke et al. 2019, Giguet-Covex et al. 2019, Parducci et al. 2017). Typically, plant sedDNA is retrieved by a metabarcoding approach that utilises the g/h primers that amplify a short fragment of the trnL P6 loop of the plant chloroplast genome (Taberlet et al. 2007). The P6 loop markers have been widely applied for the identification of plants in lake sediment samples and other environmental samples, such as soils (Edwards et al. 2018), permafrost (Willerslev et al. 2014, Zimmermann et al. 2017, Zimmermann et al. 2017) and mock samples (Pornon et al. 2016, Lamb et al. 2016). For lake sediments, the use of P6 loop markers allows an identification of plants to a low taxonomic level and the recognition of more taxa from the local lake catchment (Niemeyer et al. 2017). As plant diversity obtained from lakes' sedDNA reflects local plant communities, it is more suitable to reconstruct the past local plant diversity. It will help to reconstruct local changes of the environment and improve future predictions. Until now, we have only a limited understanding of how environmental conditions influence the preservation of the plant sedDNA proxy (Giguet-Covex et al. 2019) which requires larger sedDNA datasets spanning a wide range of environmental conditions to be investigated. The underlying modern dataset is a large sampling set covering a wide range of environmental conditions, which will provide an openly accessible data resource which can be used to address the effect of the environment on the preservation of the sedDNA proxy resulting in: improved site selection for plant sedDNA, analogues for the effects of DNA preservation and refined reconstruction of past plant communities and environmental conditions.

## Project description

### Title

Plant diversity from sedimentary DNA in Siberian and Chinese lakes

### Study area description

Lakes are located in the Siberian Arctic and in low- to high-elevation lakes from Northern China and from the Tibetan Plateau. Lake sites include large lakes, which were formed during past glacial periods and smaller lakes formed by thermokarst.

### Design description

The lake sites in Siberia were accessed during field trips conducted by the Alfred Wegener Institute in the years 2005 to 2016. The lake sites in China were visited from 2003 to 2018. This data-set has been established to correlate modern genetic plant diversity with modern vegetation mappings and climate and environmental data. The modern sedDNA data will be used to a.) evaluate suitable localities for sedDNA approaches, b.) provide analogues for preservation conditions and c.) conduct reconstruction of plant diversity and climate change mainly across glacial/interglacial phases in the Late Pleistocene–Holocene and during recent environmental change in the Anthropocene.

### Funding

The CAS Strategic Priority Research Program supported the sample collection on the Tibetan Plateau in 2018 (CAS, Grant No. XDA20090000). Jian Ni, Xianyong Cao and Kai Li were also supported by the Grant No. XDA20090000 and the China Scholarship Council (CSC). Further, fieldwork on the Tibetan Plateau was supported by the Research program (STEP) of the Chinese Academy of Science (CAS; Grant No. 2019QZKK0202), the Strategic Priority Research Program of Chinese Academy of Sciences, Pan-Third Pole Environment Study for a Green Silk Road (Pan-TPE) (XDA20040000) and the National Natural Science Foundation of China (41877459). The National Natural Science Foundation of China (NSFC) and the German Research Foundation (DFG; Grant No. 41861134030) supported the project as well. The expedition to Sakha in 2007 (07-SA) was funded by the grant from the Ministry of Nature Protection of the Republic of Sakha (Yakutia) “Bioindication assessment of the quality of drinking water of the Anabar River" (No. 8.27, 2005-2007). The Kolyma expedition 2008 (08-KO) was partly supported by the contractual theme of the Ministry of Nature Protection of the Republic of Sakha (Yakutia). The Tiksi expedition in 2009 (09-Tik) was supported by the Russian-German Biological Monitoring (BioM) research network in the terrestrial Arctic, funded by the German Ministry of Education and Research (BMBF) and supported by the German Research Foundation (DFG). The expeditions to Chatanga in 2011 and 2013 (11-CH, 13-TY) were co-financed by a Project of the Ministry of Education of the Russian Federation “NEFU Development Program: Activity 2.8 Biomonitoring of the tundra ecosystems of the North-East of Russia under the conditions of global climate change and intensification of the anthropogenic process (monitoring, ecology, paleogeography, model and environmental management technologies). The expeditions to the Omoloy region in 2014 (14-OM) and Chukotka in 2016 (16-KP) were funded by the Ministry for Education and Science of the Russian Federation (project 5.184.2014/K).

## Sampling methods

### Study extent

**Figure 1.**
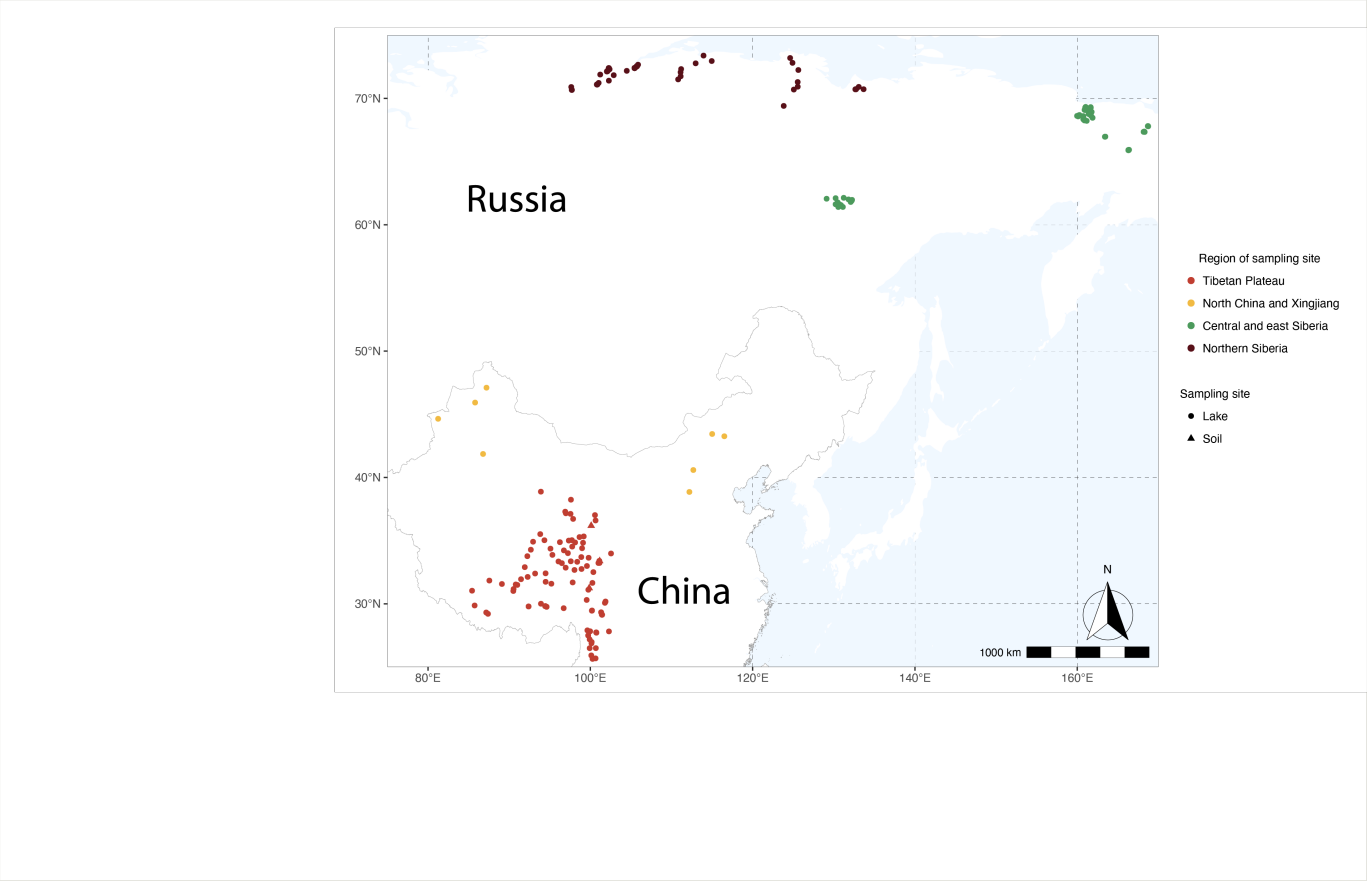
Sampling localities in Siberia and China. Lake surface-sediments or soils are indicated with a circle or triangle, respectively.

Sampling localities comprise lakes from Northern (which include expeditions to 07-SA, 09-Tik, 11-CH,13-TY, 14-OM), Eastern (08-KO, 16-KP) and Central Siberia (05-Yak), Northern and Central China and Tibet (Fig. 1). Lake physio-chemical parameters vary between the sampled lakes: water depth ranges from 0.15­–78.9 m, pH varies from 4.7–10.24 and electrical conductivity from 5–57194 m S/cm.

### Sampling description

Between the years 2003 and 2018, several expeditions were undertaken in which surface samples from 262 different localities in Siberia and China were sampled. Samples were taken with a bottom sampler from a boat mostly in the centre of the lakes (259 samples) or from soil on a lake’s shoreline (three samples).

### Quality control

The first centimetre of the surface sediment (259 samples) and shoreline soil (three samples) was carefully sampled by using gloves and single-use plastic spoons. Samples were transferred into sterile Whirl-Pak® or sterilised Nalgene tubes and were kept dark and cool (+4°C) until further treatment in the laboratories for environmental and ancient DNA at Alfred Wegener Institute Helmholtz Centre for Polar and Marine Research.

## Geographic coverage

### Description

The lake localities cover a large geographical, climatic and ecological gradient, including elevated lakes from 0 up to 5168 m a.s.l. Latitude ranges between 25°N and 73°N and longitude between 81°E and 161°E. The climatic gradient ranges in mean annual temperature between -15°C and +18°C and in mean annual precipitation between 36­ and 935 mm. The localities in Siberia span a large vegetational gradient including tundra, open woodland and boreal forest. Lake localities in China include alpine meadow, shrub, forest and steppe and also cultivated areas.

### Coordinates

25° and 73° Latitude; 81° and 161° Longitude.

## Taxonomic coverage

### Description

The retrieved DNA metabarcoding data provide 623 unique plant sequences which mainly include terrestrial and aquatic vascular plants and a few mosses. Plant DNA sequences are identified to different taxonomic levels. About 78% of sequences types are assigned to species level, 13% to genus-level and 9% to higher taxonomic levels (sub-family, family or order).

## Temporal coverage

### Notes

Sampling of lake surface-sediments was conducted in the years 2003–2018.

## Usage licence

### Usage licence

Creative Commons Public Domain Waiver (CC-Zero)

## Data resources

### Data package title

Environmental and plant diversity data from modern sedimentary DNA of lakes in Siberia and China

### Resource link

Environmental data can be retrieved at Pangaea https://doi.org/10.1594/PANGAEA.920866. DNA sequence data can be retrieved at Dryad https://doi.org/10.5061/dryad.k6djh9w4r.

### Number of data sets

2

### Data set 1.

#### Data set name

Environmental data of lake localities

#### Data format

Table

#### Number of columns

25

#### Description

Compilation of environmental data for the 262 investigated localities, which include additional intra-lake localities taken within three large lakes, namely: 16-KP-01-L02 (nine samples), 16-KP-03-L10 (five samples), 16-KP-04-L19 (four samples) (Suppl. material 1). The table includes information about the geographic coordinates, elevation, type of sample material, geographic region, water depth (at which samples were taken), pH, water conductivity, mean annual precipitation (MAP), mean annual temperature (MAP), July and January mean temperature, vegetation type and dominant plant taxa (‘dominant_Taxon1’: indicates the most frequent taxon, ‘Taxon2-10’: Taxa listed in descending order by their area of distribution in modern vegetation). If no dominant taxon is listed, the surrounding vegetation is too diverse to determine dominant taxon. 'NA' – data not available.

**Data set 1.** 

Column label	Column description
No.	Running number of items in the table
Sample name	Name of the lake locality
Latitude (N)	Latitude in decimal degrees (°N)
Longitude (E)	Longitude in decimal degrees (°E)
Elevation (m)	Elevation of lake locality in m a.s.l.
Sample type	Type of material sampled: "Lake" indicates surface sediment from within the lake, "Soil" indicates soil sampled from lake's shoreline
Geographic region	Geographic region of the lake locality
Water depth (m)	Lake water depth at sampling site
pH	pH values of the lake locality
Water Conductivity (µS/cm)	Water conductivity of the lake locality
Annual mean precipitation (mm)	Annual mean precipitation of the lake locality
Annual mean temperature (℃)	Annual mean temperature of the lake locality
July mean temperature (℃)	July mean temperature of the lake locality
January mean temperature (℃)	January mean temperature of the lake locality
Vegetation type	Vegetation type in the lake catchment
Dominant_Taxon1	Dominant plant taxon in the lake's catchment
Taxon2	Second dominant plant taxon in the lake's catchment
Taxon3	Third dominant plant taxon in the lake's catchment
Taxon4	Fourth dominant plant taxon in the lake's catchment
Taxon5	Fifth dominant plant taxon in the lake's catchment
Taxon6	Sixth dominant plant taxon in the lake's catchment
Taxon7	Seventh dominant plant taxon in the lake's catchment
Taxon8	Eighth dominant plant taxon in the lake's catchment
Taxon9	Ninth dominant plant taxon in the lake's catchment
Taxon10	Tenth dominant plant taxon in the lake's catchment

### Data set 2.

#### Data set name

Taxa list of plant species detected

#### Number of columns

11

#### Description

Taxa list of identified plant sequences with either a 100% match to the embl138 or arctborbryo taxonomic database (Suppl. material 2). The table contains the ‘ID’ – unique identifier for a cluster on the sequencing flow cell, ‘best_identity_arctborbryo’ ­– best identity with the arctborbryo database, ‘best_identity_embl138’ – best identity with the embl138 database, ‘scientific_name_by_arctborbryo’ – best taxa name with the arctborbryo, ‘scientific_name_by_embl138’ – best taxa name with the embl138, ‘DNA sequence’ – DNA sequence of the unique sequence type, ‘best_match_in_arctborbryo’ – accession number of the best reference entry in the arctborbryo database, ‘best_match_in_embl138’ – accession number of the best reference entry in the embl138 database, ‘count’ – total count of sequence type in the total sequencing project, ‘occurrence_in_PCR’ – total number of occurrences in the PCR samples.

**Data set 2.** 

Column label	Column description
No.	Running number of items in the table
ID	Unique identifier for a cluster on the sequencing flow cell
best_identity_arctborbryo	Best identity with the arctborbryo database
best_identity_embl138	Best identity with the embl138 database
scientific_name_by_arctborbryo	Best taxa name within the arctborbryo database
scientific_name_by_embl138	Best taxa name within the embl138 database
DNA sequence	Nucleotide sequence of the DNA sequence type detected
best_match_in_arctborbryo	Accession number of the best reference entry in the arctborbryo database
best_match_in_ embl138	Accession number of the best reference entry in the embl138 database
count	Total count of sequence type occurring in the filtered DNA sequencing data of all four sequencing runs
occurrence_in_PCR	Total number of occurrences in the PCRs

## Additional information

### Assessment of environmental data

Geographic coordinates, elevation, lake water depth, pH and electrical conductivity were measured with appropiate devices during the different field surveys. Geographic coordinates and elevation were measured with Garmin etrex devices. Lake water depth was measured with a hand echolot (Hondex PS-7) and pH and electrical conductivity were measured with a WTW multi-340i device. Annual mean temperature, mean temperature in July and January and mean annual precipitation were downloaded from WorldClim 2 (www.worldclim.org) and are based on the average climate data for the years 1970–2000 at a spatial resolution of 30 seconds (ca. 1 km^2^). The site-specific climate data was interpolated to the location area by using the R packages raster (Hijmans 2020). Vegetation around the lakes was extracted from vegetation maps from Russia, published by the Institute of Geography in 1990 and China (Zhang 2007). A digital version of the Russian map is available under: https://daac.ornl.gov/cgi-bin/dsviewer.pl?ds_id=700. We extracted dominant plant taxa around the lakes, considering that most of the lakes have small basins or less-developed hydrography networks. Vegetation information was then extracted by using the buffering function in R packages *raster* (Hijmans 2020). The site specific ring-buffer was measured between the sampling point to the furthest lake shoreline. For some sampling points from the relatively small lakes (radius < 50 m), this buffer was set to 100 m. Dominant plant taxa were extracted and categorised in: ‘dominant_Taxon1’: indicates the most frequent taxon, ‘Taxon2-10’: Taxa listed in descending order by their area of distribution in modern vegetation. If the extracted vegetation were identified as being too diverse, no dominant taxon (‘dominant_Taxon1’) could be identified. All environmental data are summarised in Suppl. material 1

### DNA extraction and NGS sequencing

DNA extraction of surface sediment samples was carried out in the molecular genetic laboratories for environmental genetics at Alfred Wegener Institute Helmholtz Centre for Polar and Marine Research. The genetic workflow using modern surface sediment samples includes DNA extraction, amplification, purification & pooling, DNA sequencing and bioinformatic analyses. For sediment DNA extractions, about 3–5 g of wet sediment and about 8-10 g of wet sediment for samples of 13-TY, were utilised. All DNA extractions were carried out by using the DNeasy PowerMax Soil Kit (Qiagen, Germany) and PowerMax Soil DNA Isolation kit (MoBio Laboratories, Inc., USA) following the manufacturer’s instructions with the following modifications: To each sediment sample in the Power Bead solution 2mg/ml proteinase K, 0.5ml dithiothreitol (DTT) and 1.2 ml C1 buffer were added, then vortexed for 10 min and incubated overnight in a rotating shaker at 56 °C. The final elution step was carried out with 1.6 ml C6 buffer. Each DNA extraction batch contained a maximum of nine samples and one extraction blank. Precautions were taken for all the experimental steps to avoid potential contaminations ([Bibr B5942169][Bibr B5942478]). We ran separate PCR set-ups for each of the extraction batches, including a PCR negative control (NTC). We produced at least two PCR replicates of each sample, which were set up on different days. For a few samples in the AGAK-5 run, we produced up to six PCR replicates. PCR was performed using the trnL g and h universal plant primers for the short and variable P6 loop region of the chloroplast trnL (UAA) intron (Taberlet et al. 2007). For internal barcoding of the samples and PCR replicates, we used modified g and h primers with a unique 8 bp barcode and NNN was added. After evaluation of PCR results, PCR products were purified with MinElute Kit (Qiagen, Germany), including PCR NTCs. The DNA concentration of the purified products was measured with a Qubit 2.0 Fluorometer (Thermo Fisher Scientific, Germany) and finally pooled equimolarly. In total, four pools were generated and resulted in four different sequencing runs. The pool, including samples from 13-TY, was prepared differently from the other PCR pools, because here we performed PCR replicates with the same barcode combination and pooled replicates before the final equimolar pooling of all PCR products. The four pools were sequenced using the external sequencing service (Fasteris SA, Switzerland). Four independent sequencing runs were performed (Table 1). Samples presented in this study derive from four different sequencing runs. The data from the individual runs were later merged into a single dataset for the final interpretation.

### Bioinformatic analysis of the genetic data

**Figure 2.**
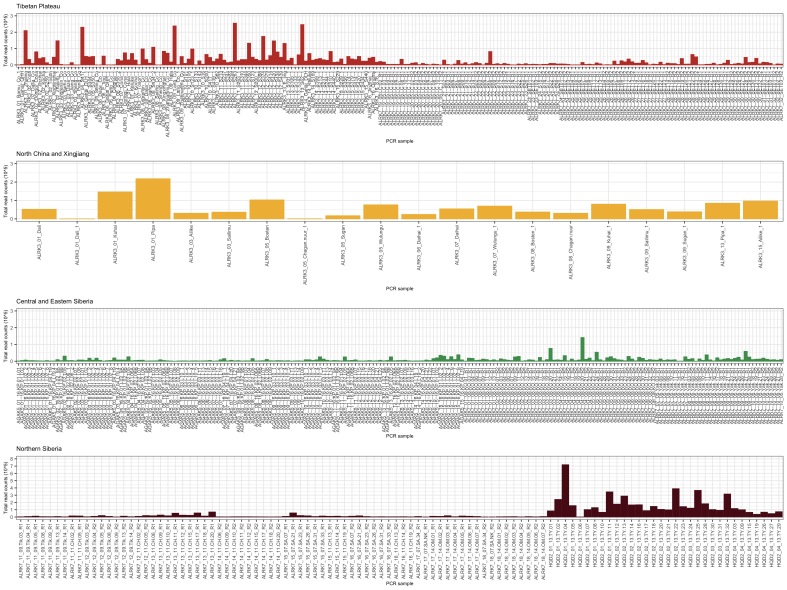
Compilation of total read counts for each PCR sample. As PCR replicates of 13-TY samples were not sequenced separately, but pooled together, only one total read count per sample is shown.

We analysed in total 262 lake samples, resulting in 688 PCRs, which divide into 553 sample PCRs and 135 PCRs of extraction blanks and NTCs. The analysis of the resulting sequence data and taxonomic assignments was done by using the OBITools package (Boyer et al. 2015). Firstly, we used *illuminapairedend* to align the raw forward and reverse reads and kept only joined reads by using *obigrep* with the option -*p 'mode!="joined*"'. Then, we used demultiplexed the joined sequences by applying *ngsfilter*, which sorts the samples according to the given unique combination of internal barcodes and the primer sequences. After demultiplexing 674 PCR samples were retrieved, while from 14 PCR samples no reads were obtained. Subsequently, we used *obigrep* to remove sequences shorter than 10 bp and *obiuniq* to merge identical reads (coverage of 100% required) by keeping the total count number for each sample in which the read was detected. Further, we used *obiclean* to remove probable PCR and sequencing errors. For taxonomic assignment, we used *ecotag* with two different databases. One database comprises a sequence reference database generated from the EMBL standard sequences (http://www.ebi.ac.uk/ena) using the release 138. To apply the EMBL database with *ecotag*, a transformation of the EMBL into an *eco*PCR database was required. Therefore, we first ran a simulated *in silico* PCR (Ficetola et al. 2010) with the g/h primers on the EMBL Standard Nucleotide Database (release 138) allowing five mismatches between the primers and target sequences. The resulting *eco*PCR output was filtered by *obigrep* to ensure assignation to the taxonomic levels: species, genus and family. Then, *obiuniq* was used to de-replicate redundant sequences and *obiconvert* was applied to transform the *eco*PCR output into an *eco*PCR database, which is finally used by *ecotag*. The second database is a sequence reference database for Arctic and Boreal vascular plants (arctborbryo database), containing 1664 vascular plant taxa and 486 bryophyte species (Soininen et al. 2015, Sønstebø et al. 2010, Willerslev et al. 2014) already adjusted to the *eco*PCR database format. For further analyses, we kept only those sequence reads that match 100% to the one or the other reference database. The total read sequence for each sample and its replicate(s) are plotted in Fig. 2. The full list of sequence types abundances is given in Suppl. material 3.

### General results of genetic data analyses

After merging raw paired-end reads and demultiplexing according to the internal barcode, two sample PCRs from lakes 16-KP-02-L08 and 16-KP-04-L22, as well as seven BLANKs and five NTCs yielded no read counts and were discarded from the dataset. After taxonomic assignment of the remaining 674 PCRs, we identified a total of 15,754,779 reads which had a 100% match to either the EMBL or arctborbryo database. For 99.6% of the reads (15,692,844 read counts), we identified 621 unique plant sequences types, while 0.39% (61,835 read counts) of the reads were assigned to non-plant taxa, including bacteria, algae and higher eukaryotic taxa (in total 72 unique sequence types). Further, we identified 340,346 reads (2.1% of the total dataset) in extraction blanks and NTCs, whereof 38.7% (23,975 reads) of reads in the BLANKs and NTCs were of non-plant origin. Amongst the samples, excluding BLANKs and NTCs, we found large differences in sample read counts which range between 1 and 718,279.

## Supplementary Material

DB66053C-AB5A-588A-B269-EA9795742CD9 10.3897/BDJ.8.e57089.suppl1 Supplementary material 1 Environmental data of lake localities Data type Lake environmental data Brief description Compilation of environmental data for the 262 investigated localities, which include additional intra-lake localities taken within three large lakes namely: 16-KP-01-L02 (nine samples), 16-KP-03-L10 (five samples), 16-KP-04-L19 (four samples). The table includes information about the geographic coordinates, elevation, type of sample material, geographic region, water depth (at which samples were taken), pH, water conductivity, mean annual precipitation (MAP), mean annual temperature (MAT), July and January mean temperature, vegetation type and dominant plant taxon (‘dominant_Taxon1’: indicates the most frequent taxon, ‘Taxon2-10’: taxa listed in descending order by their distribution area in modern vegetation). If no dominant taxon is listed, the surrounding vegetation is too diverse to determine dominant taxon. ‘NA’ – data not available. File: oo_456694.txt https://binary.pensoft.net/file/456694 Kathleen R. Stoof-Leichsenring, Sisi Liu, Weihan Jia, Kai Li, Luidmila A. Pestryakova, Steffen Mischke, Xianyong Cao, Xingqi Liu, Jian Ni, Ulrike Herzschuh

87B698CD-C244-5185-928D-C4EBB37719A2 10.3897/BDJ.8.e57089.suppl2 Supplementary material 2 Taxa list of identified plant sequences Data type Taxonomic list of plant species derived from metabarcoding Brief description A taxa list of identified plant sequences with either a 100% match to the embl138 or arctborbryo taxonomic database. The table contains the ‘ID’ – unique identifier for a cluster on the sequencing flow cell, ‘best_identity_arctborbryo’­ – best identity with the arctborbryo database, ‘best_identity_embl138’ – best identity with the embl138 database, ‘scientific_name_by_arctborbryo’ – best taxa name within the arctborbryo database, ‘scientific_name_by_embl138’ – best taxa name within the embl138 database, ‘DNA sequence’ – Nucleotide sequence of the DNA sequence type detected, ‘best_match_in_arctborbryo’ – accession number of the best reference entry in the arctborbryo database, ‘best_match_in_embl138’ – accession number of the best reference entry in the embl138 database, ‘count’ – total count of sequence type in the total sequencing project. The scientific name 'PACMAD clade' indicates the taxonomic assignment to a true group of grasses (Poaceae). 'root' indicates that the DNA sequence could not be assigned to a reference in the appropriate database. ‘occurrence_in_PCR’– total number of occurrences in the PCR samples. File: oo_457386.txt https://binary.pensoft.net/file/457386 Kathleen R. Stoof-Leichsenring, Sisi Liu, Weihan Jia, Kai Li, Ulrike Herzschuh

557ED3D8-8275-5562-AFCE-B89A3B0415DF 10.3897/BDJ.8.e57089.suppl3 Supplementary material 3 Taxa list of identified plant sequences and occurrences Data type Occurrences Brief description A taxa list of identified plant sequences with either a 100% match to the embl138 or arctborbryo taxonomic database. The table contains the ‘ID’ – unique identifier for a cluster on the sequencing flow cell, ‘best_identity_arctborbryo’ ­– best identity with the arctborbryo database, ‘best_identity_embl138’ – best identity with the embl138 database, ‘scientific_name_by_arctborbryo’ – best taxa name within the arctborbryo database, ‘scientific_name_by_embl138’ – best taxa name within the embl138 database, ‘DNA sequence’ – Nucleotide sequence of the DNA sequence type detected, ‘best_match_in_arctborbryo’ – accession number of the best reference entry in the arctborbryo database, ‘best_match_in_embl138’ – accession number of the best reference entry in the embl138 database, ‘count’ – total count of sequence type in the total sequencing project. The scientific name 'PACMAD clade' indicates the taxonomic assignment to a true group of grasses (Poaceae). 'root' indicates that the DNA sequence could not be assigned to a reference in the appropriate database. Samples are sorted according to the four sequencing runs, which is indicated by the short name of the sequencing run at the beginning of the sample name. Sample batches (which include samples, BLANKs and NTCs) belonging together are numbered. Sample names that include A and B were sediment samples taken from the same bulk surface samples and share the same environmental data as given in Suppl. material 1. File: oo_456687.txt https://binary.pensoft.net/file/456687 Kathleen R. Stoof-Leichsenring,Sisi Liu, Weihan Jia, Kai Li, Ulrike Herzschuh

## Figures and Tables

**Table 1.** Summary of NGS sequencing runs. ^1^ Sample number is equivalent to number of pooled PCR products, which also include PCR replicates of samples and corresponding BLANKs and NTCs. ^#^ Additional samples were pooled to the run, but do not belong to this project. ^§^ Sample replicates were pooled prior to final pooling of all PCR products.

**Name sequencing run**	**Device**	**Output**	**Cluster (PF)**	**Sample locations**	**Sample number^1^**
HQD-2	HiSeq	2x125bp	51'487'592	Northern Siberia (13-TY)	40^#§^
ALRK-3	NextSeq	2x150bp	26'800'628	North China, Xingijang, Tibet	153^#^
AGAK-5	NextSeq	2x150bp	87'831'867	Eastern Siberia (16-KP)	161
ALRK-7	NextSeq	2x150bp	10'20­­2'054	Northern Siberia (07-SA,09-Tik,11-CH, 14-OM), Eastern Siberia (08-KO), Central Siberia (Yak-05), North China, Xingijang, Tibet	334^#^
